# Supplementary material for: Synthesis and Luminescence Properties of Core-Shell-Shell Composites: SiO2@PMDA-Si-Tb@SiO2 and SiO2@PMDA-Si-Tb-phen@SiO2
Source: Nanomaterials (Basel). 2019 Feb 2;9(2):189. doi: 10.3390/nano9020189 (PMC6410162; doi:10.3390/nano9020189)
Supplement: Supplementary file 1 [file nanomaterials-09-00189-s001.pdf]

Supporting Information

Synthesis and Luminescence Properties of Core-Shell-Shell Composites: SiO_2@PMDA-Si-Tb@SiO_2 and SiO_2@PMDA-Si-Tb-phen@SiO_2

Lina Feng [1], Wenxian Li [1,*], Jinrong Bao [1], Yushan Zheng [2], Yilian Li [1], Yangyang Ma [1], Kuisuo Yang [1], Yan Qiao [1] and Anping Wu [1]

[1] Inner Mongolia Key Laboratory of Chemistry and Physics of Rare Earth Materials, School of Chemistry and Chemical Engineering, Inner Mongolia University, Hohhot 010021, China; nmgfln@163.com (L.F.); jinrongbao@imu.edu.cn (J.B.); yilian005@126.com (Y.L.); ma1034537689@163.com (Y.M.); nmyangks@163.com (K.Y.); 15714848705@163.com (Y.Q.); 15548098722@163.com (A.W.)

[2] Inner Mongolia Autonomous Region Food Inspection Test Center, Hohhot 010010, China; nmgzys@126.com

* Correspondence: nmglwx@163.com; Tel: +86-471-4990061

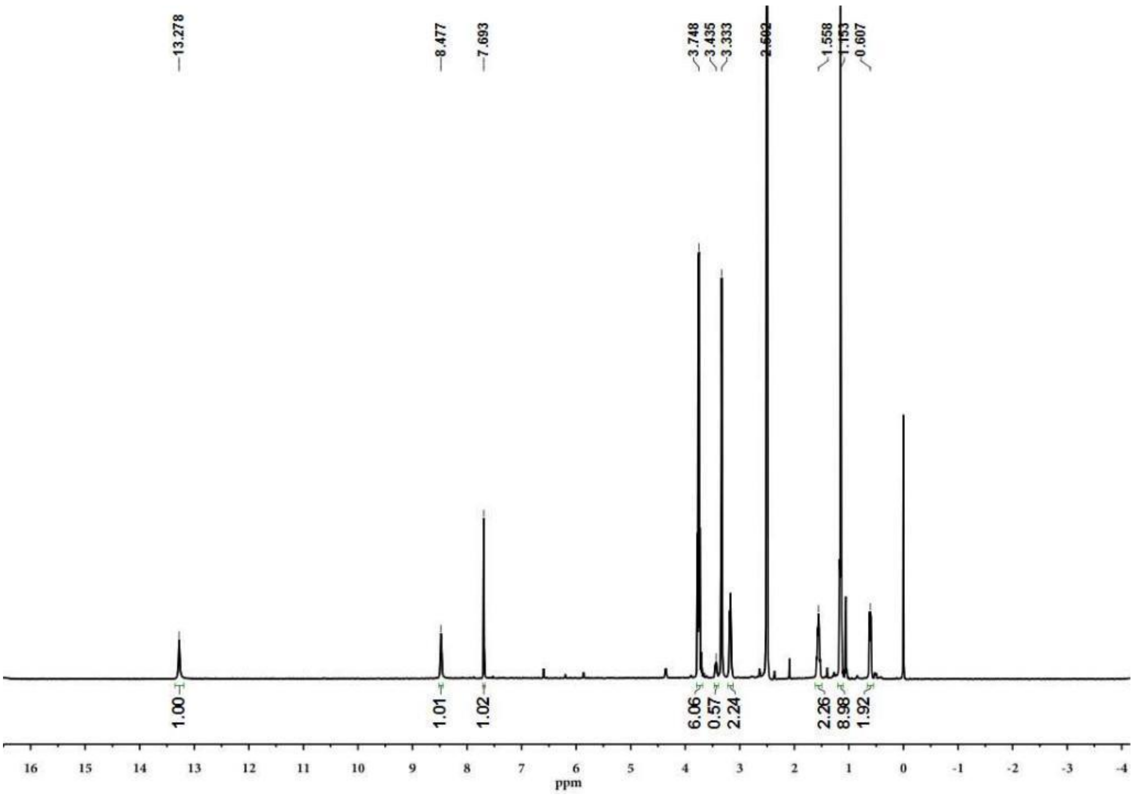

Figure S1. The ^{1}H-NMR of PMDA-Si.

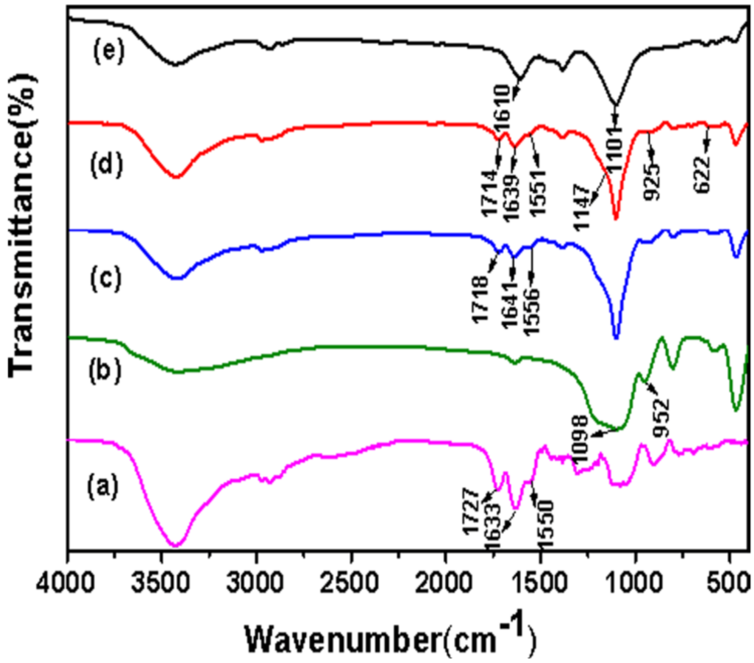

Figure S2. FT-IR spectra of PMDA-Si (**a**), SiO_2 (**b**), SiO_2@PMDA-Si (**c**), SiO_2@PMDA-Si-Tb (**d**), and SiO_2@PMDA-Si-Tb@SiO_2 (**e**).

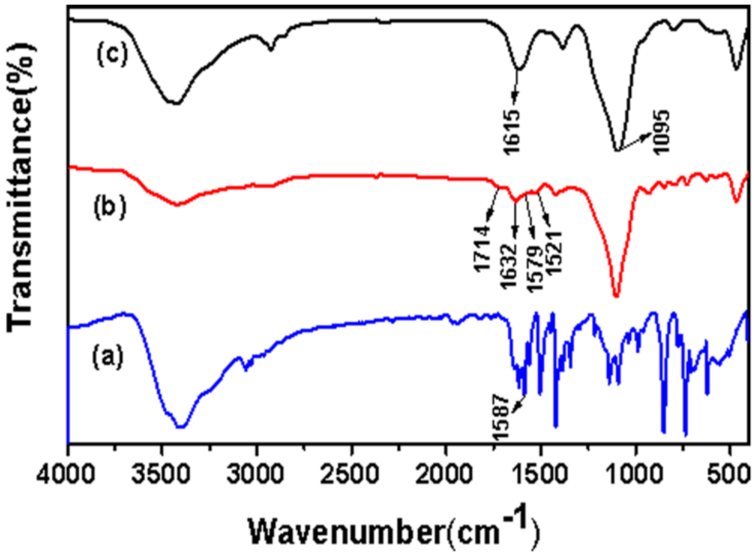

Figure S3. FT-IR spectra of phen (**a**), SiO_2@PMDA-Si-Tb-phen (**b**), and SiO_2@PMDA-Si-Tb-phen@SiO_2 (**c**).

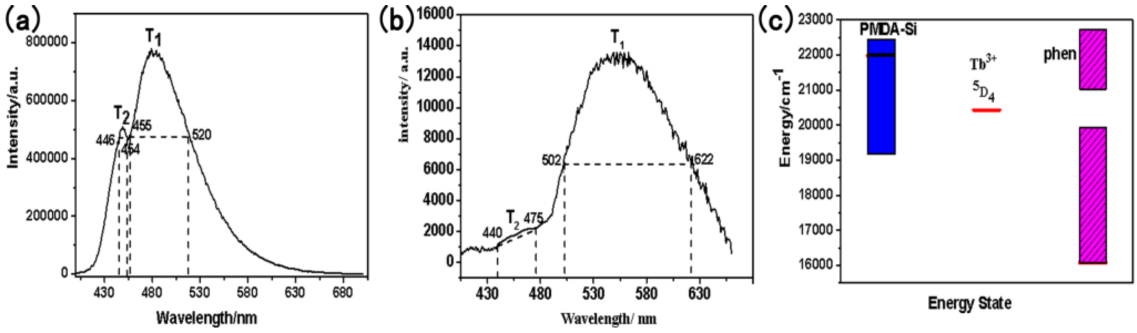

Figure S4. Phosphorescence spectra of PMDA-Si (**a**) and phen (**b**), Triplet state of PMDA-Si, phen and the excited state of Tb(III) (**c**).
